# Detecting Pilot's Engagement Using fNIRS Connectivity Features in an Automated vs. Manual Landing Scenario

**DOI:** 10.3389/fnhum.2018.00006

**Published:** 2018-01-25

**Authors:** Kevin J. Verdière, Raphaëlle N. Roy, Frédéric Dehais

**Affiliations:** ISAE-SUPAERO, Institut Supérieur de l'Aéronautique et de l'Espace, Université Fédérale de Midi-Pyrénées, Toulouse, France

**Keywords:** fNIRS, passive brain-computer-interface, classification, functional connectivity, wavelet coherence, engagement

## Abstract

Monitoring pilot's mental states is a relevant approach to mitigate human error and enhance human machine interaction. A promising brain imaging technique to perform such a continuous measure of human mental state under ecological settings is Functional Near-InfraRed Spectroscopy (fNIRS). However, to our knowledge no study has yet assessed the potential of fNIRS connectivity metrics as long as passive Brain Computer Interfaces (BCI) are concerned. Therefore, we designed an experimental scenario in a realistic simulator in which 12 pilots had to perform landings under two contrasted levels of engagement (manual vs. automated). The collected data were used to benchmark the performance of classical oxygenation features (i.e., Average, Peak, Variance, Skewness, Kurtosis, Area Under the Curve, and Slope) and connectivity features (i.e., Covariance, Pearson's, and Spearman's Correlation, Spectral Coherence, and Wavelet Coherence) to discriminate these two landing conditions. Classification performance was obtained by using a shrinkage Linear Discriminant Analysis (sLDA) and a stratified cross validation using each feature alone or by combining them. Our findings disclosed that the connectivity features performed significantly better than the classical concentration metrics with a higher accuracy for the wavelet coherence (average: 65.3/59.9 %, min: 45.3/45.0, max: 80.5/74.7 computed for HbO/HbR signals respectively). A maximum classification performance was obtained by combining the area under the curve with the wavelet coherence (average: 66.9/61.6 %, min: 57.3/44.8, max: 80.0/81.3 computed for HbO/HbR signals respectively). In a general manner all connectivity measures allowed an efficient classification when computed over HbO signals. Those promising results provide methodological cues for further implementation of fNIRS-based passive BCIs.

## 1. Introduction

It is largely admitted that pilot error represents a major cause of aircraft crashes (Li et al., 2001), being more frequently cited than mechanical failure. Safety statistics show that the progressive introduction of automation in the cockpit since the 1960's has improved safety, with modern “computerized” cockpits taking pride in an accident rate half that of the previous generation of aircraft. However, it appears that such technologies have created a new category of potentially deadly incidents whereby crews are unable to comprehend the situation presented before them, and persevere in erroneous decision-making (Dehais et al., 2010, 2015). This is especially true for the final approach and landing phases that represent almost half the on-board accidents and fatal accidents (Myers and Arnold, 2016).

Without question the development of automation has dramatically changed the role of the crew from “direct (manual) controllers” to “system supervisors/decision makers.” Both increased trust in automation and complexity of these computerized systems (Sarter et al., 1997; Dehais et al., 2012; Tessier and Dehais, 2012) reduce crew's basic flying abilities, and leave them ill equipped to cope with emergency situations when automation fails (Mumaw et al., 2001). Another drawback of automation is that it imposes long periods of inactivity and thus dramatically decreases pilot's vigilance (Wright and McGown, 2001). For instance, some recent surveys disclosed that 56% of British Airways pilots experienced sleep while on duty (Steptoe and Bostock, 2012; Reis et al., 2013). These operational situations show that automation can vary pilot's engagement from a very low engagement state (disengagement) that induces states of low vigilance and mind wandering, to a very high engagement state (over-engagement) yielding to perseveration and attentional tunneling (Wickens and Alexander, 2009). These extreme cognitive states may jeopardize safety and advocate for the introduction of monitoring solutions.

The idea of introducing physiological data into Human Machine Interface, called “Physiological Computing” (Fairclough, 2008) could allow the system to take the operator's states into account. Brain monitoring techniques such as passive Brain Computer Interfaces (pBCI) have shown their ability to detect and characterize several operator's mental state such as workload, fatigue or more generally engagement (Zander et al., 2010; Zander and Kothe, 2011; Khan and Hong, 2015; Roy and Frey, 2016). Building a system capable of doing a continuous monitoring or detecting some operator's degraded states would potentially permit it to adapt to this change to optimize both safety and performance. Such kinds of closed-looped systems are the ultimate goal for neuroadaptative technology.

While the real-time identification of these degraded mental states still remains a challenge, a first reasonable step is to characterize the brain activity when flying with and without the use of automation. One possible solution to meet this goal is to consider the use of functional near infra-red spectroscopy (fNIRS). Less popular in the BCI community than electro-encephalography (Cutini and Brigadoi, 2014), mainly due to its low temporal resolution, this brain imaging technique presents several advantages for ecological settings as its signal is less affected by electrical and motion artifacts. Moreover, its high spatial resolution allows to give a direct access to specific brain structures without additional computational costs as long as cortical areas are concerned. Thus, several studies have shown the potential of fNIRS to infer several mental states under laboratory settings or ecological settings such as flight simulators (Ayaz et al., 2012; Gateau et al., 2015).

Classically, the authors used the relative variation of local HbO and HbR concentration and related features (e.g., slope, area under the curve, skewness) to relate cerebral activation to specific cognitive tasks (Tai and Chau, 2009; Durantin et al., 2015; Gateau et al., 2015). Yet the goal is always to improve the estimation, especially in critical settings. A solution proposed by some authors is to use connectivity measures (Borghini et al., 2014) to account for brain dynamics (for a review on functional connectivity see Bastos and Schoffelen, 2015). Indeed, cognition cannot be reduced to activation of specialized brain areas but should rather been seen as the cooperation among large scale distributed neural networks (Siegel et al., 2012; Hutchison et al., 2013; van den Heuvel and Sporns, 2013). In other words, examining spontaneous hemodynamic fluctuations can provide us a great picture of the functional architecture of the brain (Fox and Raichle, 2007)

Moreover, connectivity features have been used with success to estimate various mental states based on EEG data (Roy and Frey, 2016) in laboratory settings as well as in ecological settings. For instance, a recent study combined EEG connectivity analysis and crew monitoring in simulator and showed differences in connectivity patterns during different flight phases (Toppi et al., 2016). A few studies combined optical brain imaging like fNIRS with connectivity analysis (Lu et al., 2010; Funane et al., 2011; Cui et al., 2012; Molavi et al., 2012; İşbilir et al., 2016) either to identify brain dynamics or brain-to-brain relationship yet they did not perform mental state estimation. Hence, the contribution of connectivity measures for fNIRS based on mental state estimation is yet to be assessed.

Classical correlation/covariance measures were successfully used in EEG (Gevins et al., 1987), however some spontaneous oscillation observed in blood-related imaging (fNIRS and fMRI) seems to be frequency specific, especially Low Frequency Oscillation (LFO) around 0.1 Hz (Obrig et al., 2000; Tong and Frederick, 2010). Knowing this, frequency specific connectivity metrics such as coherence and also wavelet coherence were used, which has gained some momentum in fNIRS signal analysis (Rowley et al., 2006; Cui et al., 2012; Holper et al., 2012; Mirelman et al., 2014).

The objectives of the present study are: (i) to evaluate the feasibility to estimate the pilot's engagement using fNIRS connectivity measures in an ecological setting such as a flight simulator. Secondly: (ii) to assess the potential of connectivity measures to better characterize engagement than classical measures.

To meet these goals, a simplified task was designed whereby pilots had to perform different manual and automated landings. Parieto-occipital areas were targeted as they play a key role for visual attention, particularly involved while flying (Dehais et al., 2016). Prefrontal cortex activity was also measured as its activation reflects mental demands (Gateau et al., 2015; Moro et al., 2016) and top down regulation. Off-line classification was performed over different classical metrics (average, peak, variance, skewness, kurtosis, area under curve and slope) and connectivity metrics to identify the most predictive ones. Regarding connectivity features, classical dependency measures such as: Covariance, Pearson's correlation (Greenblatt et al., 2012), Spearman's correlation (Spearman, 1904) and some spectral measures: magnitude squared coherence (Mandel and Wolf, 1976) and the wavelet coherence (Torrence and Compo, 1998; Lachaux et al., 2002; Grinsted et al., 2004) were compared (for review on connectivity metrics see Lachaux et al., 2002; Greenblatt et al., 2012).

## 2. Materials and methods

### 2.1. Participants

Twelve visual flight rules (VFR) pilots (11 males, mean group age 24 ± 3) completed the experiment. Pilots had normal or corrected-to-normal vision, normal hearing, and no psychiatric disorders. They all had medical clearance to fly. After providing written informed consent, they were instructed to complete a 5-min task training. Typical total duration of a subject's session (informed consent approval, practice task and real task) was about 1 h. This work was approved by the local ISAE-SUPAERO committee (Approval Number: CERNI-Université fédérale de Toulouse-2017-057).

### 2.2. Experimental design

**Figure 1:**
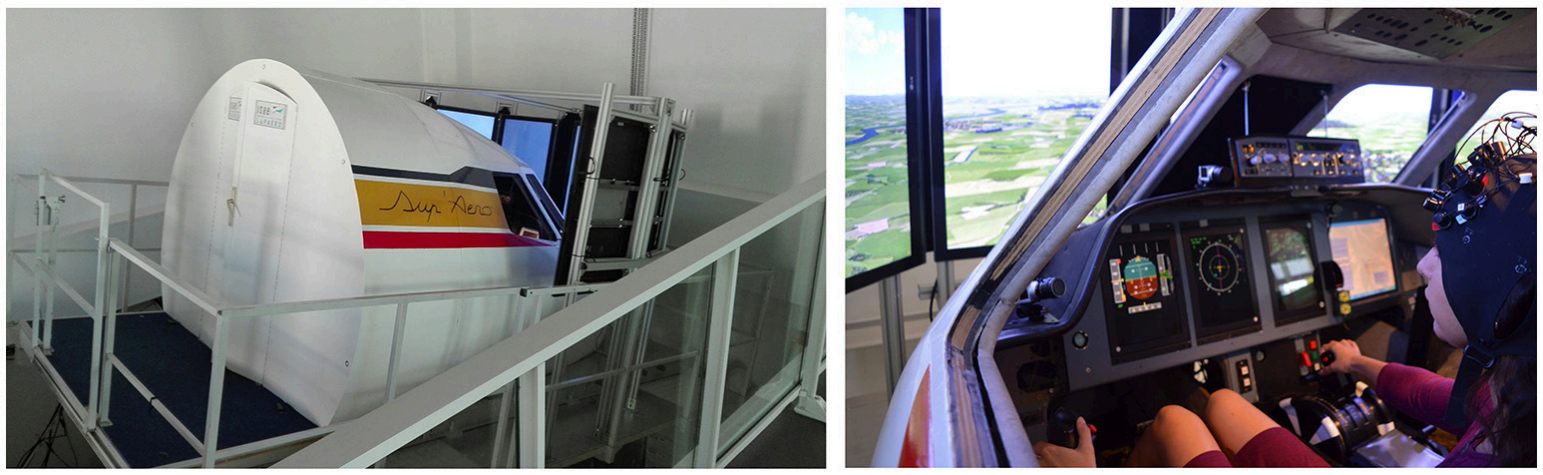
Airbus A320 twin-engine simulator at ISAE-SUPAERO. Pictures used with written consent.

The protocol consisted in 8 scenarios in a flight simulator: 4 in manual landing and 4 in automated landing. The Airbus A320 full motion simulator at ISAE-SUPAERO (French Aeronautical Engineering School in Toulouse) was used to conduct the experiment in ecological conditions. It simulates a twin-engine aircraft flight mode. The user interface is composed of a Primary Flight Display (PFD), a Navigation Display and an upper Electronic Central Aircraft Monitoring Display page. The pilot also had a Flight Control Unit (FCU) to interact with the autopilot (Figure 1).

**Figure 2:**
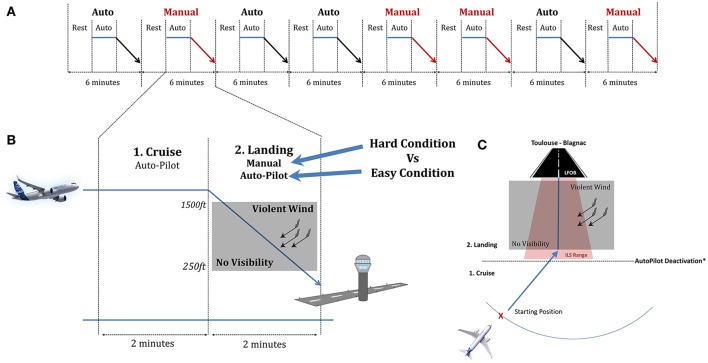
Structure of the experimental session. **(A)** Overall Flow of the experiment with the 8 scenarios. **(B)** Detailed trial for the cruise and landing phases. **(C)** Upper view of the plane trajectory. The starting position was pseudo randomly placed on the blue arc. The blackline delimited the cruise from the landing phase where the pilot deactivate or not the autopilot regarding the condition.

The scenarios were divided into 3 phases: a rest phase, a cruise phase and lastly a landing phase, which were performed either in manual mode (i.e., hard condition) in which they control the aircraft speed and trajectory, or with the autopilot engaged (easy condition; Figure 2). Landing conditions (Auto vs. Manual) were pseudo-randomly distributed.

During the cruise phase, the autopilot was engaged and the pilots were asked to relax. This phase was mostly set to serve as a baseline. When approaching the ILS (Instrument Landing System) range (approximately 2 min) they were asked either to let the autoflight system perform the landing or to disengage the automation to manually land the aircraft. Autopilot and auto throttle deactivation was done by pushing a red button on the flight stick and the throttle respectively. Participants did not know in advance whether the landing would be automated or manually executed. Considering the whole spectrum of the landing task, our experimental conditions were designed to be contrasted in terms of mental demands. The landing phase ended 10 s after the pilots touched down on the landing ground. Before starting the experiment, the participants performed a 30-min training session to familiarize themselves with the simulator environment.

### 2.3. Data acquisition

#### 2.3.1. Subjective workload assessment

After the end of the experiment, the pilots were asked to complete a commonly used subjective workload level questionnaire, the NASA-TLX (Hart and Staveland, 1988) in order to compare the two conditions. This questionnaire combines 6 factors, i.e., mental demand, physical demand, temporal demand, overall performance, frustration level, and effort.

#### 2.3.2. fNIRS recording

**Figure 3:**
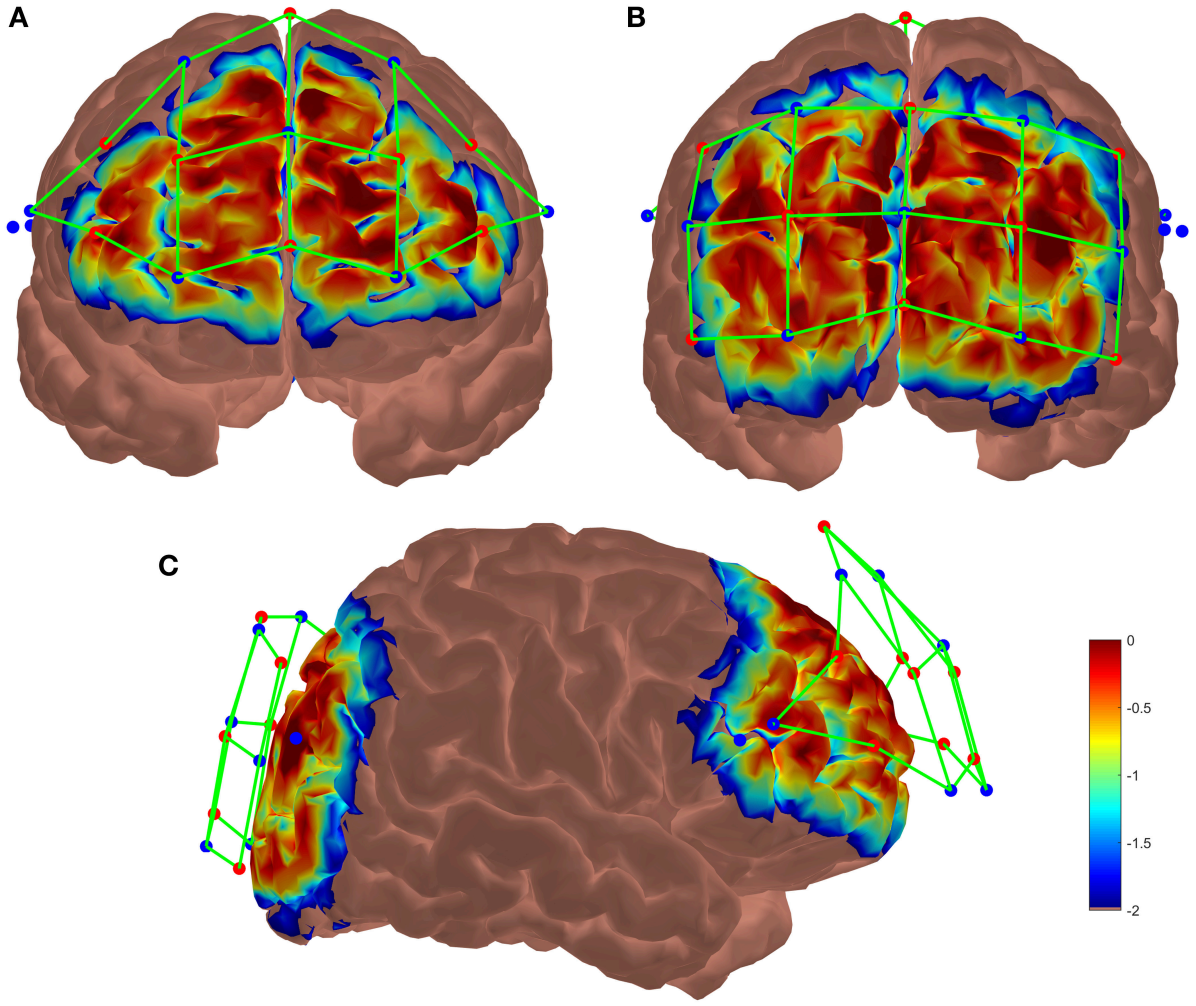
Results of the Monte-Carlo simulation over **(A)** the frontal cortex, **(B)** the occipital cortex and **(C)** both cortices from a lateral view. Red dots represent the LED emitters, blue dots the photoreceptors and green lines the channels. The colorbar unit represents the spatial sensitivity of the fNIRS measurements. It is expressed in mm^−1^ and values range from 0.01 to 1 in log10 units: −2 to 0.

Two NIRSport acquisition devices (NIRx Medical Technologies) were used in tandem mode to increase the number of sensors. Each system has 8 sources and 8 detectors receiving wavelength at 760 and 850 nm recorded at 7.8125 Hz. By using 2 systems, Frontal and Occipital areas were both covered with 8 sources and 8 detectors constrained mechanically by a plastic spacer at the appropriate distance (3 cm maximum), resulting in 42 optodes or channels. The probabilistic path of photon through cortex were estimated using the Monte-Carlo transport software tMCimg via the Atlas Viewer from Homer2 (Boas et al., 2002; Aasted et al., 2015). The optodes placement and the results of the simulation are shown Figure 3. Before starting the experiment a calibration was performed in order to check each optode's signal quality.

### 2.4. Data analysis

#### 2.4.1. Pre-processing

**Figure 4:**
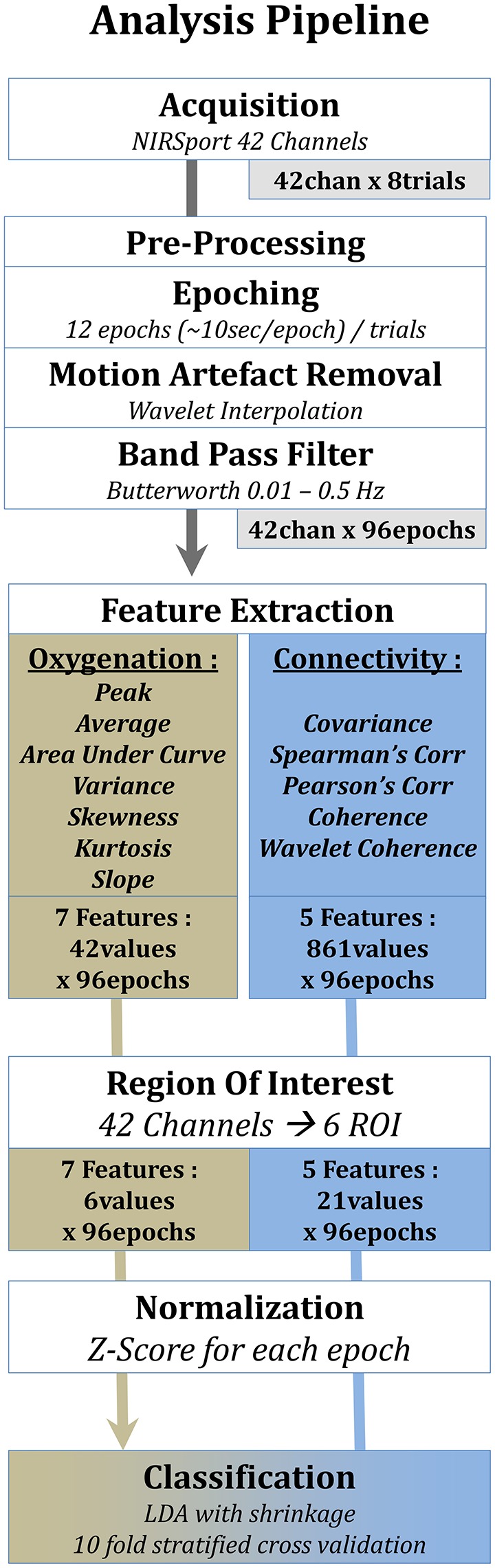
Pilot's engagement classification pipeline.

FNIRS data were analyzed using Matlab R2015b with several functions from the Homer2 software package (Dubb and Boas, 2016). The overall analysis pipeline is described in Figure 4. The landing phase was divided into epochs of 200 samples (~25 s) overlapping by 60 samples (~7.5 s). As the landing duration (152 ± 22 s) could slightly differ among participants depending on their performance, the fixed number of extracted epochs was based on the shortest landing in duration, resulting in 12 epochs per landing and per subject.

Each epoch was processed independently in order to potentially extend our method to online processing. Raw data were converted to optical densities; an artifact removal algorithm and a band pass filter were applied on each epoch separately. A wavelet interpolation method was used for the artifact correction (Molavi and Dumont, 2012). This method has been shown to have the greatest signal to noise ratio among the current artifact removal methods available (Brigadoi et al., 2014). A butterworth high pass filter (cutoff: 0.01 Hz - order 3) and a low pass filter (cutoff: 0.5 Hz - order 5) were applied for the band pass filtering step.

The filtered and artifact free data were then converted to oxy-hemoglobin [HbO] and deoxy-hemoglobin [HbR] concentration variations.

For further analysis, only the 80 centered samples (~10 s) of each epoch were kept by applying a boxcar function. This window was applied to avoid spectral leakage, specifically from the wavelet transform, and to obtain a 10 s window without overlap. At the end of this processing stage, for each landing (trial) we had 12 non-overlapping, filtered and artifact free epochs of 80 samples.

#### 2.4.2. Oxygenation measures

Oxygenation measures were computed using both the [HbO] and [HbR] signals on each epoch separately, where *x* represents either the [HbO] and [HbR] signal for one epoch (80 samples) and one optode. Seven oxygenation measures were computed (Peak, Mean, Variance, Kurtosis, Skewness, Area Under the Curve, and Slope).

The peak (maximum) and the 4th moment (average, variance, skewness, and kurtosis) were computed as follows:

(1) $$Average(x)=E(x)\;Var(x)=E[{\left(x-E(x)\right)}^{2}]$$

(2) $$Skew(x)=\frac{E[{\left(x-E(x)\right)}^{3}]}{\left(E{\left[{\left(x-E(x)\right)}^{2}\right]}^{3/2}\right)}\;Kurt(x)=\frac{E[{\left(x-E(x)\right)}^{4}]}{\left(E{\left[{\left(x-E(x)\right)}^{2}\right]}^{2}\right)}$$

The Area Under the Curve (AUC) was calculated by summing the absolute values of the signal.

(3) AUC=∑|x|

The slope was computed using the least-squared linear regression with the polyfit matlab function.

#### 2.4.3. Connectivity measures

Connectivity measures were computed, as previously, using both the [HbO] and [HbR] signals on each epoch separately, where *x* and *y* represents two signals from two different channels. Five oxygenation measures were computed (Covariance, Pearson's correlation, Spearman's correlation, Coherence, and Wavelet Coherence).

Covariance of two signals *x* and *y* can be described as a “measure of joint variability”:

(4) COV(x,y)=E(x−E(x))×E(y−E(y))

Where *E* represents the expected value. Intuitively, covariance characterizes the simultaneous variations of two signals. Covariance will be positive when the differences between the signals and their averages tend to be of the same sign and tend to be negative in the opposite case.

Pearson's correlation coefficient is the covariance of two signals normalized by the product of their standard deviation (std). It represents the linear correlation between two signals, its values ranges from −1 to +1 meaning respectively a linear negative and positive correlation and 0 corresponding to no correlation at all.

(5) $$Pearson(x,y)=\frac{COV(x,y)}{std(x)\times std(y)}$$

Spearman rank correlation coefficient is “defined as the Pearson correlation coefficient between the ranked variable” (Myers and Arnold, 2003).

(6) $$Spearman(x,y)=\frac{COV(rg_{x},rg_{Y})}{std(rg_{x})\times std(rg_{y})}$$

Where *rg*_*x*_ and *rg*_*y*_ are the ranked variable (of *x* and *y* respectively). Using the rank instead of the values allows describing monotonic non-linear relationship between signals where the Pearson's coefficient only characterizes linear relationship.

Spectral Coherence *C*_*xy*_(*f*) or Magnitude squared Coherence is defined as the absolute squared value of the cross-spectral density of two signals (*x* and *y*) for a frequency f, normalized by the product of their auto-spectral density:

(7) $$C_{xy}(f)=\frac{|G_{xy}(f){|}^{2}}{G_{xx}(f)\times G_{yy}(f)}$$

Where *G*_*xy*_(*f*) represents the cross spectral density (being the spectrum of the cross correlation function) for a frequency *f*. *G*_*xx*_(*f*) and *G*_*yy*_(*f*) being the auto spectral density (i.e., the spectrum of the auto correlation function) respectively for *x* and *y*. Spectral coherence can be seen as a correlation coefficient in the frequency domain.

For the last one, a coherence measure based on the wavelet transform was used (Torrence and Compo, 1998): the wavelet coherence. The wavelet coherence power $R_{n}^{2}\left(s\right)$ can be defined as:

(8) $$R_{n}^{2}(s)=\frac{|S(s^{-1}W_{n}^{xy}(s){|}^{2}}{S(s^{-1}|W_{n}^{x}(s){|}^{2})S(s^{-1}|W_{n}^{y}(s){|}^{2})}$$

Where $W_{n}^{x}\left(s\right)$ and $W_{n}^{y}\left(s\right)$ represent respectively the wavelet transform of *x* and *y* at the *n* time point for a wavelet scale *s*. $W_{n}^{xy}\left(s\right)$ is the cross wavelet transform of *x* and *y* (being the wavelet transform of the cross correlation function). *S* is a smoothing operator (for more detail see Torrence and Compo, 1998).

This measure can be seen as “a localized correlation coefficient in time frequency space” (Grinsted et al., 2004). Coherence values range from 0 to 1, 1 meaning there is a perfectly phase-locked oscillations at a given frequency for the two analyzed signals.

Connectivity measures were computed on each epoch separately and for each couple of channels namely $C\left(\begin{array}{c} n\\ k \end{array}\right)$ = 861 couples (*k* = 2, *n* = 42). At this step, we had 42 measures for each oxygenation feature and 861 measures for each connectivity feature per epoch and per subject.

### 2.5. Data classification

#### 2.5.1. Feature extraction

##### 2.5.1.1. Region of interest

In order to reduce the amount of data and the dimensionality, the 42 different channels were combined into 6 regions of interest (ROI): Frontal-Left and Right, Fronto-Central; Occipital-Left and Right and Occipito-Central.

For the oxygenation features, it was done by averaging all the features from channels included in these 6 differents regions. For the connectivity features, 15 possible connections were possible across the 6 ROI. Values were firstly evaluated for each pair (861 couples) and then averaged across couples connecting the same regions. Couples of channels included inside one ROI were also kept, which gave 15 + 6 = 21 connectivity measures.

At this step, we had 6 measures for each oxygenation feature and 21 measures for each connectivity feature per epoch and per subject.

##### 2.5.1.2. Frequency specific measures

For the two coherence measures (Magnitude Squared Coherence and Wavelet Coherence), the obtained coherence values were averaged for a frequency range between 0.3125 Hz (1/3.2 s) and 0.08 Hz (1/12.8 s) accordingly to the fNIRS literature (Cui et al., 2012).

##### 2.5.1.3. Normalization: z-score

Regarding all features, they were normalized by z-scoring (i.e., transform it to have 0 mean and 1 standard deviation; Toronov et al., 2001; Tsunashima and Yanagisawa, 2009; Sasai et al., 2011).

#### 2.5.2. Classification and cross-validation

A Linear Discriminant Analysis (LDA) with regularization of the empirical covariance matrix by shrinkage, also known as “shrinkage method”, was used (Friedman, 1989; Blankertz et al., 2011). This method has proved its robustness for BCI and passive BCI (pBCI) application (Roy et al., 2016a,b) but also with fNIRS (Herff et al., 2013; Bauernfeind et al., 2014; Hennrich et al., 2015).

Our paradigm was an intra-subject binary classification. Each subject performed 8 landings (4 of each of the 2 conditions). Data were processed to obtain 12 10 s epochs for each landing which gives 12 × 8 = 96 epochs (examples) for each subject. Our model prediction performance was assessed by using a stratified cross validation, which is a good tradeoff between bias and variance estimation (Kohavi and Sommerfield, 1995; Friedman et al., 2001). The classifier was trained with examples that originated from 6 different landings (3 of each of the 2 conditions, i.e., 6^*^12 = 72 examples) and tried to predict examples from the last 2 landings (1 of each condition, i.e., 2^*^12 = 24 examples). This method was applied for every combination (16) of landings left out of the training set and the averaged performance was kept.

Regarding the features, 2 types of comparisons were done. Firstly, a single feature comparison where each feature classification performance is assessed separately was performed. Secondly, features were merged together to evaluate their potential. They were combined 2 by 2 and the classification performance obtained with each couple was assessed.

#### 2.5.3. Statistical assessment

##### 2.5.3.1. Subjective workload comparison

A paired-sample *t*-test was performed in order to compare the average overall workload obtained for the 2 conditions among subjects.

##### 2.5.3.2. Classification performance significance

For a 2-class problem like ours, the theoretical chance level for classification is 100/2 = 50 %, but this is only right for an infinite sample number. To assess the significance of our classifier (decoding accuracy) the classification error was modelized by a binomial cumulative distribution (see Combrisson and Jerbi, 2015 for more details):

(9) $$P(Z)=\sum_{i=z}^{n}\left(\begin{array}{c} n\\ k \end{array}\right)\times {\left(\frac{1}{c}\right)}^{i}\times {\left(\frac{c-1}{c}\right)}^{n-1}$$

Where - *P* is the probability to predict the correct class at least Z times - *n* the number of samples - *c* the number of classes.

The performance of our classification pipeline was assessed by repeating the stratified cross validation 16 times and averaging it. As stated earlier, our classifier was trained with 72 samples and tested on the last 24 samples. By using the cumulative binomial distribution, it sets the 5% significance classification threshold at 58.3%.

##### 2.5.3.3. Classification performance comparison

In order to compare the classification performance for each feature, a repeated measure ANOVA was used considering FEATURES (or FEATURES COUPLE) and CHROMOPHORE (HbO/HbR) as within factors. A *post-hoc* Tukey's Honestly Significant Difference (HSD) procedure was applied to perform multiple comparisons.

## 3. Results

### 3.1. Subjective workload assessment

Participants rated their workload significantly higher for the manual landing condition (*M* = 66.6 ± 9) than the automatic landing condition (*M* = 18.7 ± 7; *t*(11) = −17.43, *p* < 10^−8^).

### 3.2. Classification with individual features

**Figure 5:**
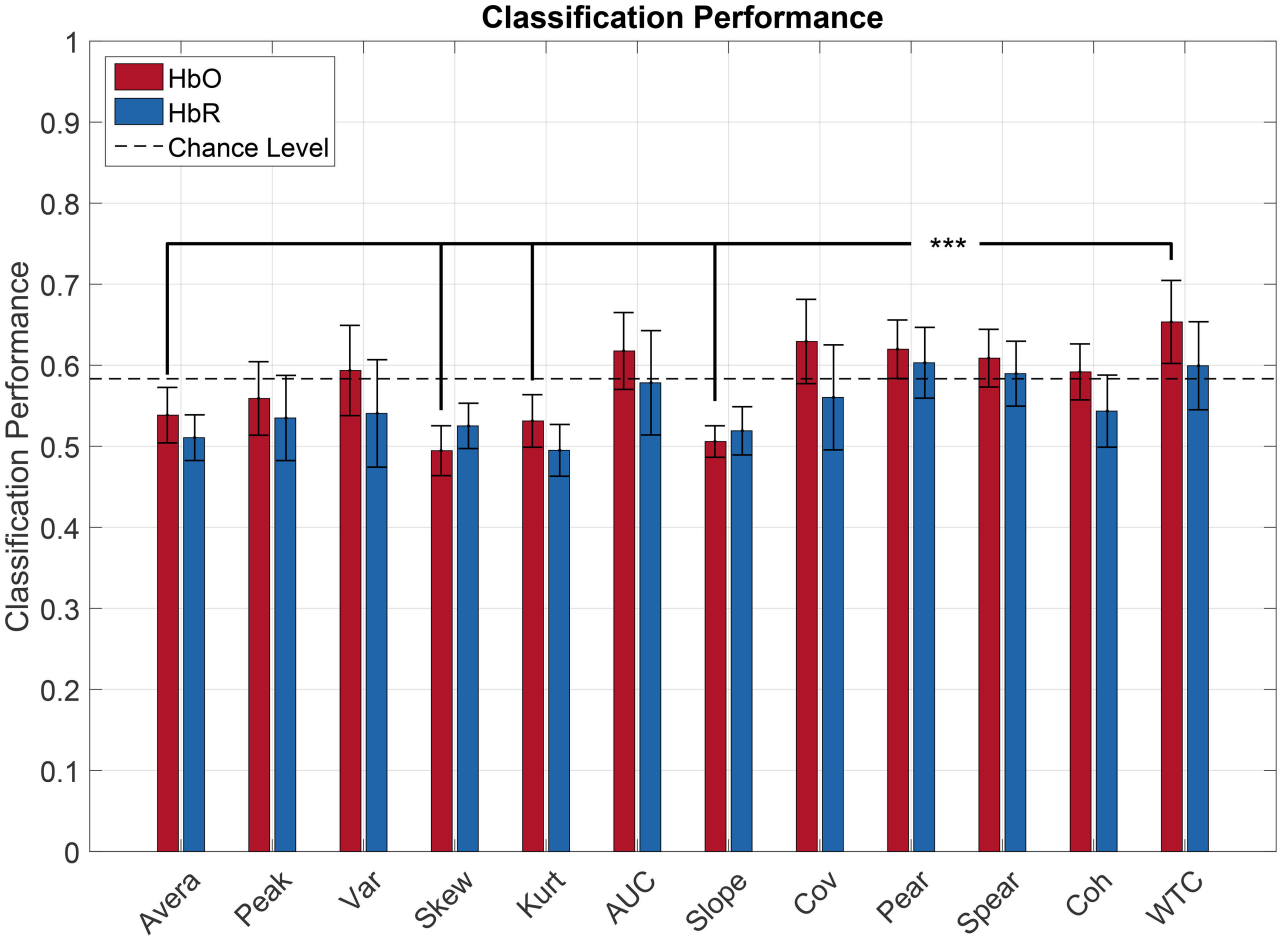
Pilot's engagement classification performance function of the type of fNIRS-based feature (average across subject). Blue and red bars represents features extracted from respectively [HbR] and [HbO] signals. Error bars represents the confidence interval at 95 %. The black lines indicate the most relevant significant effect for our research question (^***^p < 0.05).

Figure 5 illustrates the classification performances for each feature computed over the HbR and HbO signals. In order to compare classification performance among features, a repeated measure ANOVA was done.

The statistical analysis showed that there was a significant effect of feature type on classification performance [$F_{\left(11,121\right)}=5.66,p<10^{-3}$] and it also revealed a significant effect of the chromophore used [*F*_(1, 11)_ = 8.73, *p* < 0.05]. *Post-hoc* comparisons revealed significant differences among features mainly for HbO. In particular, Wavelet Coherence had a significantly better performance than the Average, Skewness, Kurtosis, and Slope. Also, every connectivity feature gave a significantly greater performance than the Skewness. Moreover, regarding HbR, the Wavelet Coherence and the Covariance gave a significantly greater performance than the Kurtosis. All the connectivity features did not exhibit significant differences between one another. *Post-hoc* comparisons did not show any significant effect of the chromophore on the classification performance regardless of the feature used. In other words, every feature gave non-significant different results when using either the HbO or HbR signals for the classification.

Moreover, every connectivity feature computed over the HbO signals led to an average classification performance above chance level (>58.3 %). Furthermore, Pearson's, Spearman's correlation, and the Wavelet Coherence exceeded the chance level for both HbO and HbR. Concerning classical oxygenation features, the AUC and Variance were the only features to reach a classification performance above chance level but only when computed over HbO signals.

Regarding the best features, Wavelet Coherence benefited of the best classification performance among subjects with an average 65.34 and 59.94% of good classification respectively for HbO and HbR. The second was the Covariance (62.93 and 56.03 %) followed by the Area Under the Curve (61.76 and 57.83%) for HbO and HbR respectively.

### 3.3. Classification with combined features

**Figure 6:**
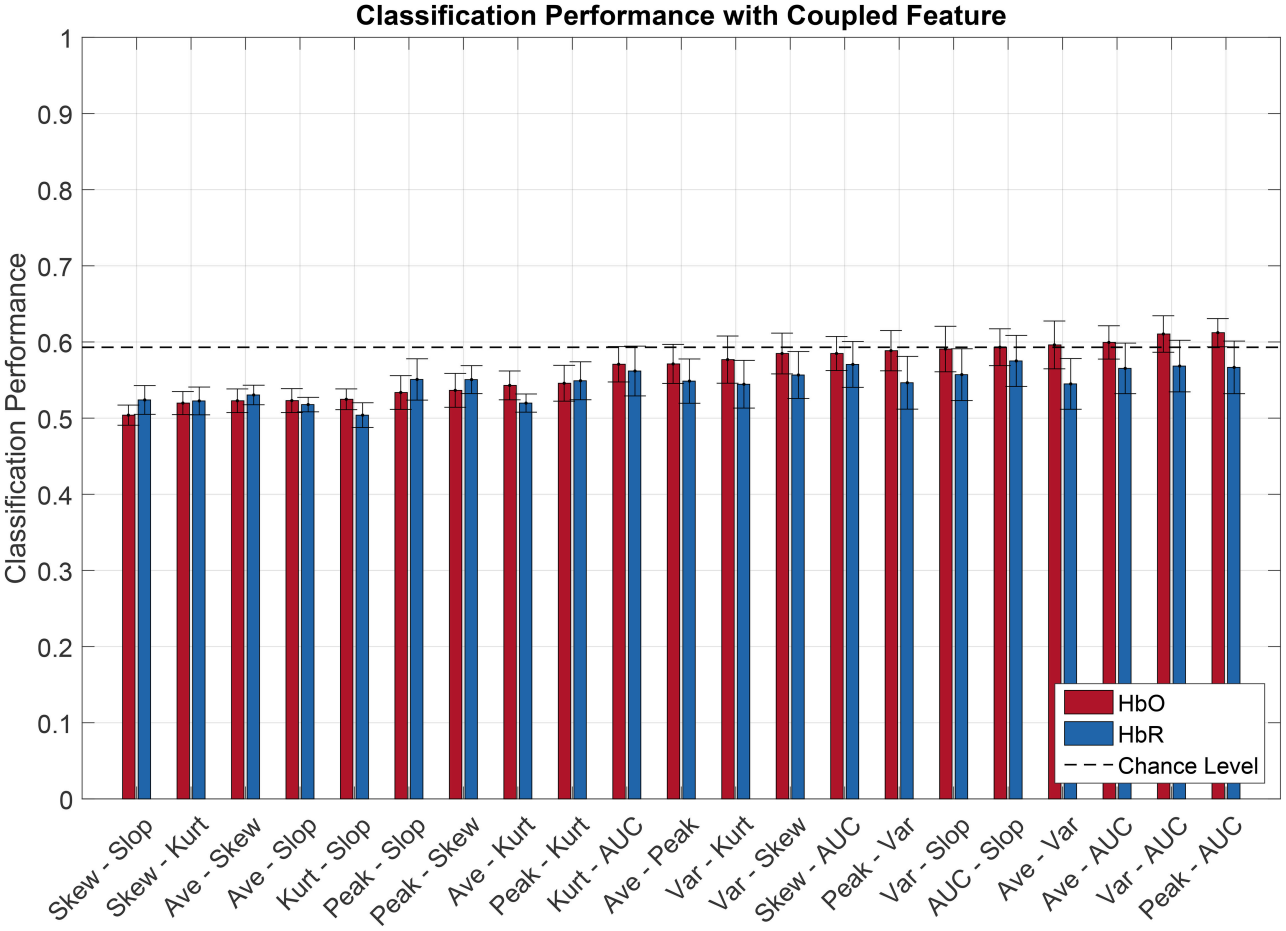
Pilot's engagement classification performance function of couple fNIRS-based oxygenation feature used (average across subject). Blue and red bars represents features extracted from respectively [HbR] and [HbO] signals. Error bars represents the confidence interval at 95 %.

**Figure 7:**
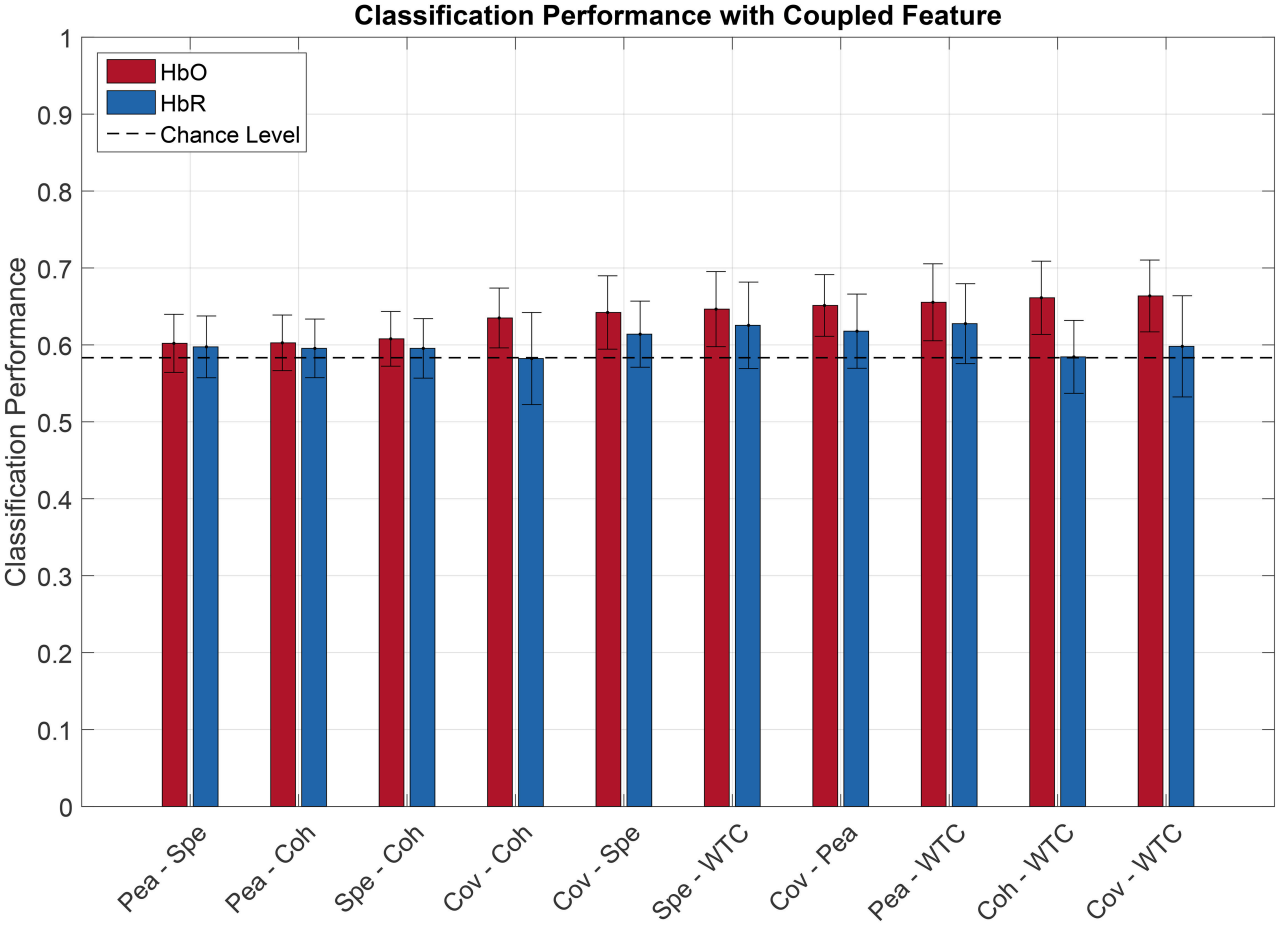
Pilot's engagement classification performance function of couple fNIRS-based connectivity feature used (average across subject). Blue and red bars represents features extracted from respectively [HbR] and [HbO] signals. Error bars represents the confidence interval at 95 %.

Figures 6, 7 show the averaged classification performance for all the possible combinations of 2 oxygenation or 2 connectivity features respectively.

Following the same procedure as before, a repeated measure ANOVA was done with the data showed Figures 6, 7. It revealed that there was a significant effect of the feature couple [$F_{\left(30,\;330\right)}=5.42,p<10^{-3}$] but not of the chromophore [*F*_(1, 11)_ = 2.47, *p* = 0.14] on the classification performance.

When evaluating multiple comparisons for HbO, the main observation is that the 7 best connectivity couples gave a significantly greater classification performance than the 7 worse oxygenation couples. Besides that, it can also be noted here that connectivity couples did not exhibit significant differences between one another.

For oxygenation features, 9 out of 21 couples of features led to a classification performance above chance level, namely AUC-Peak, AUC-Variance, AUC-Average, Average-Variance, AUC-Slope, Variance-Slope, Peak-Variance, AUC-Skewness, and Variance-Skew. The AUC-Peak couple reached a classification performance of 61.2 and 56.7% for HbO and HbR respectively. Moreover AUC is in 5 of these 9 best couples. Regarding combined connectivity features, every connectivity couple reached a classification performance above chance except the couple Covariance-Coherence when computed over HbR. The best couple (Covariance-WaveletCoherence) led to a classification performance of 66.4 and 59.8% (for HbO and HbR respectively).

Results for every feature couple, including couples mixing oxygenation and connectivity features, for every subject are given in Tables 1, 2.

**Table 1:** Classification performance (HbO/HbR) for every possible combination of 2 features with an average performance across subjects **under chance level** (<58.3 %) computed over HbO.

**Features**	**S1**	**S2**	**S3**	**S4**	**S5**	**S6**	**S7**	**S8**	**S9**	**S10**	**S11**	**S12**	**Average**
Skew - Slo	40/55	46/53	46/47	57/56	50/58	58/64	50/57	51/49	51/45	52/51	55/39	49/56	50/52
Skew - Kurt	57/57	45/54	53/48	54/57	48/65	55/51	61/51	55/55	50/48	47/47	55/39	43/56	52/52
Ave - Skew	54/53	58/51	48/51	64/51	48/62	52/63	52/51	51/52	56/49	43/51	56/49	47/54	52/53
Ave - Slo	61/57	59/51	48/47	57/51	52/51	56/59	41/49	52/51	51/53	47/52	51/49	53/52	52/52
Kurt - Slo	60/53	49/49	53/47	47/55	52/55	57/53	60/51	57/53	49/53	46/44	49/36	50/56	52/50
Peak - Slo	56/55	60/52	42/39	55/57	54/47	58/73	43/55	62/70	63/53	47/50	59/63	41/47	53/55
Peak - Skew	51/56	56/53	45/46	62/54	52/56	53/69	53/54	59/62	65/51	47/49	63/62	38/49	54/55
Ave - Kurt	67/50	57/53	54/49	59/54	53/51	52/57	60/47	61/55	50/59	47/43	49/53	43/53	54/52
Peak - Kurt	61/54	61/54	50/45	48/55	55/46	57/65	55/55	65/71	64/64	46/42	57/63	35/47	55/55
Kurt - AUC	69/61	57/63	60/42	52/55	60/45	63/66	60/60	65/81	61/55	53/40	45/62	40/44	57/56
Ave - Peak	65/52	64/59	50/43	62/42	60/49	59/74	49/56	65/71	68/57	46/49	60/62	38/43	57/55
Skew - Coh	66/48	51/50	50/53	65/54	51/64	46/56	69/52	62/47	58/54	54/52	59/69	56/66	57/55
Var - Kurt	67/55	64/55	62/43	41/53	64/46	57/66	64/60	74/82	64/53	52/44	45/57	38/41	58/54
Skew - Spe	58/60	49/57	49/53	68/66	64/77	57/59	66/73	62/46	61/54	48/50	49/48	66/62	58/59

*Results are rounded to the closest integers and ordered by their average value (the last couple (row) is the best performing on average across subjects). Columns refer to subjects (S1–S12) and rows to each feature couple (Ave, Average; Pea, Peak; Var, Variance; Skew, Skewness; Kurt, Kurtosis; AUC, Area Under the Curve; Cov, Covariance; Pear, Pearson's; Spear, Spearman's; Coh, Coherence; WTC, Wavelet Coherence)*.

**Table 2:** Classification performance (HbO/HbR) for every possible combination of 2 features with an average performance across subjects **above chance level** (>58.3 %) computed over HbO.

**Features**	**S1**	**S2**	**S3**	**S4**	**S5**	**S6**	**S7**	**S8**	**S9**	**S10**	**S11**	**S12**	**Average**
Var - Skew	64/56	60/52	57/46	57/51	60/60	60/70	62/59	78/82	66/45	50/51	51/54	38/42	58/56
Skew - AUC	68/60	54/57	58/46	61/53	59/56	66/71	62/59	69/80	63/50	51/48	49/63	42/41	58/57
Kurt - Coh	66/53	52/45	57/48	58/57	53/59	52/50	69/49	69/53	55/59	55/54	57/68	60/67	59/55
Slo - Coh	66/52	58/41	52/45	61/59	51/61	51/64	71/52	69/52	55/58	54/57	59/68	59/70	59/56
Peak - Var	64/50	65/52	57/40	52/50	64/41	61/72	56/56	73/81	70/55	55/55	53/63	37/41	59/55
Var - Slo	67/58	69/49	57/42	48/56	66/51	69/72	56/59	75/84	63/47	50/54	44/57	43/40	59/56
AUC - Slo	72/60	59/59	60/40	59/64	57/51	67/76	60/62	71/78	64/48	53/46	44/62	46/43	59/58
Skew - Pea	58/60	50/56	48/53	63/66	64/75	58/63	69/76	66/48	68/56	48/52	55/45	67/61	59/59
Ave - Var	66/51	67/53	59/50	59/40	64/48	64/72	57/58	80/82	69/54	47/51	46/56	39/40	60/54
Ave - AUC	70/61	58/60	61/45	61/47	57/44	67/72	60/61	70/81	66/54	53/45	49/63	46/45	60/57
Peak - Coh	65/48	58/49	53/50	66/55	54/57	56/67	70/52	65/58	64/52	54/55	60/71	54/61	60/56
Kurt - Cov	66/57	72/51	64/46	52/53	63/63	63/67	65/53	74/81	64/49	52/48	46/63	39/42	60/56
Pea - Spe	64/58	52/59	55/58	65/60	63/73	59/64	66/72	67/52	65/58	50/54	49/48	66/62	60/60
Pea - Coh	63/57	52/51	51/51	64/64	64/72	54/60	70/68	65/49	64/58	54/59	55/61	68/64	60/60
Ave - Coh	71/45	60/43	58/51	67/53	56/58	53/60	70/49	65/50	59/60	55/57	56/68	58/65	61/55
Spe - Coh	67/57	53/53	52/53	64/65	66/71	57/56	69/68	64/46	64/58	53/60	54/63	67/66	61/60
Skew - Cov	63/61	65/49	60/53	61/47	66/69	65/68	63/52	75/80	68/44	49/50	54/63	42/45	61/57
Kurt - Spe	65/57	51/58	59/56	63/67	66/70	58/56	68/72	72/51	63/56	48/48	54/49	65/60	61/58
Var - AUC	71/62	63/61	66/42	61/54	60/44	67/69	58/60	72/83	66/53	54/47	50/64	43/43	61/57
Peak - AUC	69/60	61/55	60/39	62/57	59/43	65/76	61/57	70/79	69/54	57/49	56/67	46/44	61/57
Peak - Cov	65/52	70/52	63/50	59/50	65/61	69/70	61/47	75/82	68/46	53/56	52/66	37/44	61/56
Kurt - Pea	64/57	55/55	59/56	61/65	68/71	57/59	71/74	73/53	65/55	48/50	54/47	65/60	62/59
Slo - Spe	60/58	58/53	55/51	68/61	68/70	62/66	67/72	67/49	65/55	49/55	51/47	67/61	62/58
Slo - Cov	65/58	71/41	59/52	59/53	70/63	68/71	61/53	76/84	65/45	49/53	50/65	47/43	62/57
Slo - Pea	59/59	60/51	54/49	62/59	69/70	62/70	69/74	68/52	67/55	50/53	54/47	69/61	62/58
Peak - Spe	61/62	58/56	53/55	72/62	66/71	62/66	65/72	71/64	70/55	49/52	56/57	63/55	62/60
Var - Cov	64/55	70/52	63/53	59/45	67/66	70/68	60/52	75/82	71/51	52/52	52/67	44/43	62/57
AUC - Coh	71/53	60/48	57/45	63/56	57/54	62/71	73/54	75/80	63/60	62/57	50/68	58/59	63/59
Ave - Spe	66/62	61/55	52/60	73/61	69/71	63/65	64/71	66/51	66/58	49/52	55/52	65/61	63/60
Ave - Cov	68/51	73/46	64/55	64/39	65/63	67/70	58/50	78/81	68/49	49/52	50/62	46/42	63/55
Var - Coh	70/50	65/44	59/49	61/56	58/60	58/68	72/53	76/80	66/57	58/52	56/66	53/59	63/58
Peak - Pea	63/62	60/55	52/54	67/59	66/70	60/69	68/74	74/65	71/55	51/52	59/56	63/57	63/61
AUC - Cov	69/62	67/54	66/52	64/54	67/63	68/68	60/52	76/82	69/49	54/51	52/70	43/47	63/59
Ave - Pea	66/62	59/52	52/59	70/58	69/71	61/65	69/76	68/52	68/60	51/51	55/51	69/63	63/60
Var - Spe	64/64	62/56	57/53	67/64	66/71	61/66	66/72	83/77	67/55	54/53	49/58	61/53	63/62
Skew - WTC	42/58	53/65	57/65	70/54	81/70	64/74	78/71	63/46	67/49	54/48	60/57	68/64	63/60
AUC - Spe	64/66	57/65	52/53	70/64	67/71	66/67	70/72	78/78	68/56	52/55	50/66	64/54	63/64
Cov - Coh	68/55	72/40	59/53	64/52	62/69	64/66	69/48	76/80	66/55	57/54	53/69	53/55	63/58
AUC - Pea	65/65	59/64	52/53	66/60	65/69	67/70	70/74	79/80	71/57	55/54	52/61	67/56	64/64
Cov - Spe	64/64	69/55	61/55	70/62	67/70	62/67	66/67	85/78	64/52	53/56	50/58	60/53	64/61
Kurt - WTC	52/54	58/65	60/65	67/55	80/64	66/73	76/66	67/46	65/48	56/43	61/56	65/64	64/58
Peak - WTC	53/54	64/67	52/60	66/53	82/65	66/77	76/70	65/62	68/46	55/47	60/59	65/60	64/60
Var - Pea	66/64	65/55	58/53	63/59	66/71	61/68	71/75	83/79	69/58	55/53	52/57	66/55	65/62
Slo - WTC	44/57	62/61	64/63	67/58	83/70	65/76	76/70	65/46	65/52	54/49	63/60	68/66	65/61
Spe - WTC	55/60	54/66	60/62	70/65	85/75	65/74	74/74	66/45	66/56	57/45	57/59	65/71	65/63
Cov - Pea	64/64	68/54	59/54	65/57	66/71	64/70	71/71	82/80	67/54	56/55	53/57	67/54	65/62
Ave - WTC	54/53	64/64	62/64	66/50	84/70	64/74	77/68	63/46	67/51	55/49	62/57	66/64	65/59
Pea - WTC	54/59	57/62	59/61	71/63	85/74	67/77	78/74	66/49	68/57	57/48	59/59	67/71	66/63
Coh - WTC	59/50	61/55	60/55	72/56	84/69	65/74	78/63	68/46	67/52	56/50	55/63	68/68	66/58
Cov - WTC	57/55	67/57	61/63	66/50	81/70	67/77	79/67	77/81	68/45	58/43	57/59	59/51	66/60
Var - WTC	59/53	63/63	59/63	67/51	81/66	68/80	78/70	75/83	70/48	59/43	59/57	62/53	67/61
AUC - WTC	64/59	61/64	60/61	70/53	80/63	69/81	77/70	68/78	72/48	59/45	57/64	65/55	67/62

*Results are rounded to the closest integers and ordered by their average value (the last couple (row) is the best performing on average across subjects). Columns refer to subjects (S1–S12) and rows to each feature couple (Ave, Average; Pea, Peak; Var, Variance; Skew, Skewness; Kurt, Kurtosis; AUC, Area Under the Curve; Cov, Covariance; Pear, Pearson's; Spear, Spearman's; Coh, Coherence; WTC, Wavelet Coherence)*.

## 4. Discussion

The main motivation of the present study was to assess the potential of connectivity measures to classify two different levels of task engagement with fNIRS under relatively ecological settings. We therefore designed a protocol whereby pilots had to perform several manual and automated landings. Our subjective measures confirmed that these two situations were contrasted as manual landing led to significantly higher subjective NASA-TLX scores than automated landing. Our overall classification results confirmed that the two different engagement levels could be discriminated in a flight simulator. This is in line with previous neuroergonomics studies showing that this brain optical imaging technique is well suited for mental state monitoring in ecological situations (Herff et al., 2013; Durantin et al., 2015; Gateau et al., 2015; Foy et al., 2016).

The best classification accuracy reached 66.9 %, a result that does not compare favorably with recent studies at first hand. For instance, Hong et al. (2015) obtained a classification performance of 75.6 % on 10 subjects with a mental motor imagery and mental arithmetic paradigm using average and slope features over chromophore concentration. Holper and Wolf (2011) did a complex vs. simple imaginary movement paradigm with 12 subjects. By combining different features such as the average, variance, skewness and kurtosis computed over HbO and HbR, they reached a performance of 81.3 %. Naseer et al. (2016) obtained a 93 % classification performance with almost similar features to classify mental arithmetic vs. rest on 7 subjects. However, these studies did not consider a continuous but rather an event locked assessment of a specific cognitive activity contrarily to our flying task involved different executive and attentional skills. Interestingly enough and contrary to our results, Khan and Hong (2015) showed that classical oxygenation metrics could yield to a high accuracy (84.9 %) when continuously monitoring drowsiness under ecological settings such as driving in simulated conditions. The comparison with our study remains challenging as the construct of engagement is probably more subtle to be captured. Eventually, the limited number of trials did not allow us to optimize the training of our model to guarantee high classification accuracy.

Interestingly, the connectivity measures led to better classification performance than the classical oxygenation metrics (i.e., chromophore concentration variation). The better performance of the connectivity metrics over classical ones could rely on two main explanations. Firstly, one has to consider that the analysis of task-related concentrations (i.e., hemodynamic response) is time-locked to the event. It has been proposed that these task-related responses induce a small increase (<5%) in neural energy consumption compared to the overall brain energy consumption (Raichle and Mintun, 2006). Thus by focusing only on a localized hemodynamic response, the majority of the brain activity is dismissed. It is now well admitted that cognition relies on the activation of several distributed brain areas rather than single dedicated processing units (Siegel et al., 2012; Hutchison et al., 2013; van den Heuvel and Sporns, 2013). Thus, the analysis of the interaction between neural networks provides more information on the brain dynamics, especially when concerned with the understanding of complex real-life task (Cui et al., 2012; Leff et al., 2015; İşbilir et al., 2016). Secondly, some relevant studies disclosed that frequency or amplitude correlations among spontaneous LFOs (around 0.1 Hz) are tightly linked to cortical processes (Lowe et al., 1998; Xiong et al., 1999; Obrig et al., 2000, see Siegel et al., 2012 for a review). As a matter of fact, when considering continuous monitoring of the brain activity, where no specific events are expected, connectivity features based on frequency or amplitude coupling can give an insight on the ongoing cognitive processes.

The comparison of the connectivity metrics classification performance revealed that covariance, correlation (Pearson's or Spearman's) and wavelet coherence led to significantly higher classification accuracies than respectively 3, 2, and 4 classical oxygenation metrics. It is interesting to note that the formers present complementary advantages. On one hand, correlation and covariance are straightforward and low cost computational measure to implement. This is of great advantage as long as pBCIs are concerned. On the other hand, the wavelet coherence takes into account both time and phased locked oscillations. While being used for some years by the fMRI community, the wavelet coherence metrics has only recently been applied to fNIRS signal (Cui et al., 2012; İşbilir et al., 2016). Wavelet coherence also allows to target specific and relevant frequency bands such as LFOs as discussed previously. However, the implementation of wavelet coherence based pBCIs remains challenging as this metric requires a high number of wavelet convolutions and the calculation costs could be critical in an online paradigm. One possible promising approach to overcome this issue is to consider dimensionality reduction (Guyon and Elisseeff, 2003). Taken together our findings provide some methodological guidance for the implementation of fNIRS based BCI metrics. To the best of our knowledge, this study is one of the rare to benchmark different fNIRS connectivity metrics and to use them for classification purposes in ecological settings. It paves the way toward online mental state estimation in ecological aeronautical settings, but some challenges still remain.

Despite its potential interests, our paper has several limitations. Firstly, this experiment involved 12 subjects that only performed four trials of each conditions. This limited number of trials relied on a compromise as the participants would experience fatigue and discomfort if wearing the cap for a long period (around 40 min). Secondly, the choice of the two contrasted conditions (automatic vs. manual) can be discussed since potential confounds such as motor responses could influence our measures. Yet we did not target motor areas therefore the risk is low. However, our motivation is to monitor pilots' brain activity when facing realistic flying conditions. The designing of well contrasted and controlled conditions in ecological environments such as flying remains challenging. This first experiment was meant to set the path to more refined protocols to characterize different tasks with a view to perform crew monitoring as achieved by Toppi et al. (2016). The third limitation is regarding the fNIRS signal analysis. Indeed, fNIRS signal is the result of a global component influenced by skin blood flow and a local neuronal component. Some algorithms based on spatial filtering and principal component analysis such as the one proposed by Zhang et al. (2016) could have been used if the analysis was not done on each epoch separately. Moreover fNIRS signals can also be influenced by other physiological activities such as heartbeats, respiration or changes in blood pressure. It would have been interesting to also record those activities to evaluate how they can correlate with the engagement level. Regarding the paradigm settings and because of these limitations, despite the fact that our classification performance were very high and satisfying, it is not possible to make any claim regarding the underlying neurophysiological processes.

Finally, the performance of the classification pipeline needs to be improved before its implementation in the cockpit as such rate of false negative detection cannot be afforded as it is in such critical systems, even though using multisensory fusion this accuracy level is still usable. A promising way to increase classification performance could be to use a bimodal EEG-fNIRS pBCI (Fazli et al., 2012).

## Author contributions

Study conception and design: KV, RR, FD. Data acquisition: KV, FD. Data Analysis: KV. Data Interpretation and Writing: KV, RR, FD.

### Conflict of interest statement

The authors declare that the research was conducted in the absence of any commercial or financial relationships that could be construed as a potential conflict of interest.

## References

[B1] Aasted C. M. Yücel M. A. Cooper R. J. Dubb J. Tsuzuki D. Becerra L.. (2015). Anatomical guidance for functional near-infrared spectroscopy: atlasviewer tutorial. Neurophotonics 2, 020801–020801. 10.1117/1.NPh.2.2.020801 26157991 PMC4478785

[B2] Ayaz H. Shewokis P. A. Bunce S. Izzetoglu K. Willems B. Onaral B. (2012). Optical brain monitoring for operator training and mental workload assessment. Neuroimage 59, 36–47. 10.1016/j.neuroimage.2011.06.023 21722738

[B3] Bastos A. M. Schoffelen J.-M. (2015). A tutorial review of functional connectivity analysis methods and their interpretational pitfalls. Front. Syst. Neurosci. 9:175. 10.3389/fnsys.2015.00175 26778976 PMC4705224

[B4] Bauernfeind G. Steyrl D. Brunner C. Müller-Putz G. R. (2014). Single trial classification of fnirs-based brain-computer interface mental arithmetic data: a comparison between different classifiers, in Engineering in Medicine and Biology Society (EMBC), 2014 36th Annual International Conference of the IEEE (Chicago, IL: IEEE), 2004–2007. 10.1109/EMBC.2014.6944008 25570376

[B5] Blankertz B. Lemm S. Treder M. Haufe S. Müller K.-R. (2011). Single-trial analysis and classification of erp componentsa tutorial. Neuroimage 56, 814–825. 10.1016/j.neuroimage.2010.06.048 20600976

[B6] Boas D. A. Culver J. Stott J. Dunn A. (2002). Three dimensional monte carlo code for photon migration through complex heterogeneous media including the adult human head. Opt. Express 10, 159–170. 10.1364/OE.10.000159 19424345

[B7] Borghini G. Astolfi L. Vecchiato G. Mattia D. Babiloni F. (2014). Measuring neurophysiological signals in aircraft pilots and car drivers for the assessment of mental workload, fatigue and drowsiness. Neurosci. Biobehav. Rev. 44, 58–75. 10.1016/j.neubiorev.2012.10.003 23116991

[B8] Brigadoi S. Ceccherini L. Cutini S. Scarpa F. Scatturin P. Selb J.. (2014). Motion artifacts in functional near-infrared spectroscopy: a comparison of motion correction techniques applied to real cognitive data. Neuroimage 85, 181–191. 10.1016/j.neuroimage.2013.04.082 23639260 PMC3762942

[B9] Combrisson E. Jerbi K. (2015). Exceeding chance level by chance: the caveat of theoretical chance levels in brain signal classification and statistical assessment of decoding accuracy. J. Neurosci. Methods 250, 126–136. 10.1016/j.jneumeth.2015.01.010 25596422

[B10] Cui X. Bryant D. M. Reiss A. L. (2012). Nirs-based hyperscanning reveals increased interpersonal coherence in superior frontal cortex during cooperation. Neuroimage 59, 2430–2437. 10.1016/j.neuroimage.2011.09.003 21933717 PMC3254802

[B11] Cutini S. Brigadoi S. (2014). Unleashing the future potential of functional near-infrared spectroscopy in brain sciences. J. Neurosci. Methods 232, 152–156. 10.1016/j.jneumeth.2014.05.024 24880046

[B12] Dehais F. Causse M. Vachon F. Tremblay S. (2012). Cognitive conflict in human–automation interactions: a psychophysiological study. Appl. Ergon. 43, 588–595. 10.1016/j.apergo.2011.09.004 21992968

[B13] Dehais F. Peysakhovich V. Scannella S. Fongue J. Gateau T. (2015). Automation surprise in aviation: real-time solutions, in Proceedings of the 33rd Annual ACM Conference on Human Factors in Computing Systems (Seoul: ACM), 2525–2534.

[B14] Dehais F. Roy R. N. Gateau T. Scannella S. (2016). Auditory alarm misperception in the cockpit: an eeg study of inattentional deafness, in International Conference on Augmented Cognition (Toronto, ON: Springer), 177–187.

[B15] Dehais F. Tessier C. Christophe L. Reuzeau F. (2010). The perseveration syndrome in the pilot's activity: guidelines and cognitive countermeasures, in Human Error, Safety and Systems Development, eds Palanque P. Vanderdonckt J. Winckler M. (Berlin, Heidelberg: Springer), 68–80. 10.1007/978-3-642-11750-3_6

[B16] Dubb J. Boas D. (2016). Homer 2 Toolbox, v2.1. Available online at: https://www.nitrc.org/frs/shownotes.php?release_id=3156

[B17] Durantin G. Scannella S. Gateau T. Delorme A. Dehais F. (2015). Processing functional near infrared spectroscopy signal with a kalman filter to assess working memory during simulated flight. Front. Hum. Neurosci. 9:707. 10.3389/fnhum.2015.00707 26834607 PMC4719469

[B18] Fairclough S. H. (2008). Fundamentals of physiological computing. Interact. Comput. 21, 133–145. 10.1016/j.intcom.2008.10.011

[B19] Fazli S. Mehnert J. Steinbrink J. Curio G. Villringer A. Müller K.-R.. (2012). Enhanced performance by a hybrid nirs–eeg brain computer interface. Neuroimage 59, 519–529. 10.1016/j.neuroimage.2011.07.084 21840399

[B20] Fox M. D. Raichle M. E. (2007). Spontaneous fluctuations in brain activity observed with functional magnetic resonance imaging. Nat. Rev. Neurosci. 8:700. 10.1038/nrn2201 17704812

[B21] Foy H. J. Runham P. Chapman P. (2016). Prefrontal cortex activation and young driver behaviour: a fnirs study. PLoS ONE 11:e0156512. 10.1371/journal.pone.0156512 27227990 PMC4881939

[B22] Friedman J. Hastie T. Tibshirani R. (2001). The Elements of Statistical Learning. Vol 1 Springer series in statistics. New York, NY: Springer.

[B23] Friedman J. H. (1989). Regularized discriminant analysis. J. Am. Stat. Assoc. 84, 165–175. 10.1080/01621459.1989.10478752

[B24] Funane T. Kiguchi M. Atsumori H. Sato H. Kubota K. Koizumi H. (2011). Synchronous activity of two people's prefrontal cortices during a cooperative task measured by simultaneous near-infrared spectroscopy. J. Biomed. Opt. 16:077011. 10.1117/1.3602853. 21806291

[B25] Gateau T. Durantin G. Lancelot F. Scannella S. Dehais F. (2015). Real-time state estimation in a flight simulator using fnirs. PLoS ONE 10:e0121279. 10.1371/journal.pone.0121279 25816347 PMC4376943

[B26] Gevins A. S. Morgan N. H. Bressler S. L. Cutillo B. A. White R. Illes J.. (1987). Human nueroelectric patterns predict performance accuracy. Science 235, 580–586. 10.1126/science.3810158 3810158

[B27] Greenblatt R. E. Pflieger M. Ossadtchi A. (2012). Connectivity measures applied to human brain electrophysiological data. J. Neurosci. Methods 207, 1–16. 10.1016/j.jneumeth.2012.02.025 22426415 PMC5549799

[B28] Grinsted A. Moore J. C. Jevrejeva S. (2004). Application of the cross wavelet transform and wavelet coherence to geophysical time series. Nonlinear Process. Geophys. 11, 561–566. 10.5194/npg-11-561-2004

[B29] Guyon I. Elisseeff A. (2003). An introduction to variable and feature selection. J. Machine Learn. Res. 3, 1157–1182.

[B30] Hart S. G. Staveland L. E. (1988). Development of nasa-tlx (task load index): results of empirical and theoretical research. Adv. Psychol. 52, 139–183. 10.1016/S0166-4115(08)62386-9

[B31] Hennrich J. Herff C. Heger D. Schultz T. (2015). Investigating deep learning for fnirs based bci, in Engineering in Medicine and Biology Society (EMBC), 2015 37th Annual International Conference of the IEEE (Milan: IEEE), 2844–2847. 10.1109/EMBC.2015.7318984 26736884

[B32] Herff C. Heger D. Fortmann O. Hennrich J. Putze F. Schultz T. (2013). Mental workload during n-back task—quantified in the prefrontal cortex using fnirs. Front. Hum. Neurosci. 7:935. 10.3389/fnhum.2013.00935 24474913 PMC3893598

[B33] Holper L. Scholkmann F. Wolf M. (2012). Between-brain connectivity during imitation measured by fnirs. Neuroimage 63, 212–222. 10.1016/j.neuroimage.2012.06.028 22732563

[B34] Holper L. Wolf M. (2011). Single-trial classification of motor imagery differing in task complexity: a functional near-infrared spectroscopy study. J. Neuroeng. Rehabil. 8:34. 10.1186/1743-0003-8-34 21682906 PMC3133548

[B35] Hong K.-S. Naseer N. Kim Y.-H. (2015). Classification of prefrontal and motor cortex signals for three-class fnirs–bci. Neurosci. Lett. 587, 87–92. 10.1016/j.neulet.2014.12.029 25529197

[B36] Hutchison R. M. Womelsdorf T. Allen E. A. Bandettini P. A. Calhoun V. D. Corbetta M.. (2013). Dynamic functional connectivity: promise, issues, and interpretations. Neuroimage 80, 360–378. 10.1016/j.neuroimage.2013.05.079 23707587 PMC3807588

[B37] İşbilir E. Çakir M. P. Cummins F. Ayaz H. (2016) Investigating brain-brain interactions of a dyad using fnir hyperscanning during joint sentence reading task, in 3rd International Symposium on Brain cognitive Science (Istanbul).

[B38] Khan M. J. Hong K.-S. (2015). Passive bci based on drowsiness detection: an fnirs study. Biomed. Opt. Express 6, 4063–4078. 10.1364/BOE.6.004063 26504654 PMC4605063

[B39] Kohavi R. Sommerfield D. (1995). Feature subset selection using the wrapper method: overfitting and dynamic search space topology, in KDD′95: Proceedings of the First International Conference on Knowledge Discovery and Data Mining, eds Fayyad U. Uthurusamy R. (Montréal, QC: AAAI Press), 192–197.

[B40] Lachaux J.-P. Lutz A. Rudrauf D. Cosmelli D. Le Van Quyen M. Martinerie J.. (2002). Estimating the time-course of coherence between single-trial brain signals: an introduction to wavelet coherence. Clin. Neurophysiol. 32, 157–174. 10.1016/S0987-7053(02)00301-5 12162182

[B41] Leff D. R. James D. R. Orihuela-Espina F. Kwok K.-W. Sun L. W. Mylonas G.. (2015). The impact of expert visual guidance on trainee visual search strategy, visual attention and motor skills. Front. Hum. Neurosci. 9:526. 10.3389/fnhum.2015.00526 26528160 PMC4604246

[B42] Li G. Baker S. P. Grabowski J. G. Rebok G. W. (2001). Factors associated with pilot error in aviation crashes. Aviat. Space Environ. Med. 72, 52–58. 11194994

[B43] Lowe M. Mock B. Sorenson J. (1998). Functional connectivity in single and multislice echoplanar imaging using resting-state fluctuations. Neuroimage 7, 119–132. 10.1006/nimg.1997.0315 9558644

[B44] Lu C.-M. Zhang Y.-J. Biswal B. B. Zang Y.-F. Peng D.-L. Zhu C.-Z. (2010). Use of fnirs to assess resting state functional connectivity. J. Neurosci. Methods 186, 242–249. 10.1016/j.jneumeth.2009.11.010 19931310

[B45] Mandel L. Wolf E. (1976). Spectral coherence and the concept of cross-spectral purity. JOSA 66, 529–535. 10.1364/JOSA.66.000529

[B46] Mirelman A. Maidan I. Bernad-Elazari H. Nieuwhof F. Reelick M. Giladi N.. (2014). Increased frontal brain activation during walking while dual tasking: an fnirs study in healthy young adults. J. Neuroeng. Rehabil. 11:85. 10.1186/1743-0003-11-85 24886198 PMC4055254

[B47] Molavi B. Dumont G. A. (2012). Wavelet-based motion artifact removal for functional near-infrared spectroscopy. Physiol. Meas. 33:259. 10.1088/0967-3334/33/2/259 22273765

[B48] Molavi B. Gervain J. Dumont G. A. Noubari H. A. (2012). Functional connectivity analysis of cortical networks in functional near infrared spectroscopy using phase synchronization, in Engineering in Medicine and Biology Society (EMBC), 2012 Annual International Conference of the IEEE (San Diego, CA: IEEE), 5182–5185. 10.1109/EMBC.2012.6347161 23367096

[B49] Moro S. B. Carrieri M. Avola D. Brigadoi S. Lancia S. Petracca A.. (2016). A novel semi-immersive virtual reality visuo-motor task activates ventrolateral prefrontal cortex: a functional near-infrared spectroscopy study. J. Neural Eng. 13:036002. 10.1088/1741-2560/13/3/036002 27001948

[B50] Mumaw R. J. Sarter N. Wickens C. D. (2001). Analysis of pilot's monitoring and performance on an automated flight deck, in 11th International Symposium on Aviation Psychology (Columbus, OH).

[B51] Myers J. L. Arnold D. (2003). Research Design and Statistical Analysis. Mahwah, NJ: Lawrence Erlbaum.

[B52] Myers J. L. Arnold D. (2016). Statistical Summary of Commercial Jet Airplane Accidents. Seattle, WA: Boeing.

[B53] Naseer N. Noori F. M. Qureshi N. K. Hong K.-S. (2016). Determining optimal feature-combination for lda classification of functional near-infrared spectroscopy signals in brain-computer interface application. Front. Hum. Neurosci. 10:237. 10.3389/fnhum.2016.00237. 27252637 PMC4879140

[B54] Obrig H. Neufang M. Wenzel R. Kohl M. Steinbrink J. Einhäupl K.. (2000). Spontaneous low frequency oscillations of cerebral hemodynamics and metabolism in human adults. Neuroimage 12, 623–639. 10.1006/nimg.2000.0657 11112395

[B55] Raichle M. E. Mintun M. A. (2006). Brain work and brain imaging. Annu. Rev. Neurosci. 29, 449–476. 10.1146/annurev.neuro.29.051605.112819 16776593

[B56] Reis C. Mestre C. Canhão H. (2013). Prevalence of fatigue in a group of airline pilots. Aviat. Space Environ. Med. 84, 828–833. 10.3357/ASEM.3548.2013 23926658

[B57] Rowley A. Payne S. Tachtsidis I. Ebden M. Whiteley J. Gavaghan D.. (2006). Synchronization between arterial blood pressure and cerebral oxyhaemoglobin concentration investigated by wavelet cross-correlation. Physiol. Meas. 28:161. 10.1088/0967-3334/28/2/005 17237588

[B58] Roy R. N. Bonnet S. Charbonnier S. Campagne A. (2016a). Efficient workload classification based on ignored auditory probes: a proof of concept. Front. Hum. Neurosci. 10:519. 10.3389/fnhum.2016.00519 27790109 PMC5062542

[B59] Roy R. N. Charbonnier S. Campagne A. Bonnet S. (2016b). Efficient mental workload estimation using task-independent eeg features. J. Neural Eng. 13:026019. 10.1088/1741-2560/13/2/026019 26877162

[B60] Roy R. N. Frey J. (2016). Neurophysiological markers for passive brain–computer interfaces, in Brain–Computer Interfaces 1: Foundations and Methods, eds Bougrain L. Clerc L. Lotte F. (Indianapolis, IN: Wiley Online Library), 85–100

[B61] Sarter N. B. Woods D. D. Billings C. E. (1997). Automation surprises. Handb. Hum. Fact. Ergon. 2, 1926–1943.

[B62] Sasai S. Homae F. Watanabe H. Taga G. (2011). Frequency-specific functional connectivity in the brain during resting state revealed by nirs. Neuroimage 56, 252–257. 10.1016/j.neuroimage.2010.12.075 21211570

[B63] Siegel M. Donner T. H. Engel A. K. (2012). Spectral fingerprints of large-scale neuronal interactions. Nat. Rev. Neurosci. 13:121. 10.1038/nrn3137 22233726

[B64] Spearman C. (1904). The proof and measurement of association between two things. Am. J. Psychol. 15, 72–101. 10.2307/1412159 3322052

[B65] Steptoe A. Bostock S. (2012). A Survey of Fatigue and Well-Being among Commercial Airline Pilots. London: UCL Psychobiology Group.

[B66] Tai K. Chau T. (2009). Single-trial classification of nirs signals during emotional induction tasks: towards a corporeal machine interface. J. Eeuroengi. Rehabil. 6:39. 10.1186/1743-0003-6-39 19900285 PMC2779792

[B67] Tessier C. Dehais F. (2012). Authority management and conflict solving in human-machine systems. AerospaceLab 4, 1–10.

[B68] Tong Y. Frederick B. D. (2010). Time lag dependent multimodal processing of concurrent fmri and near-infrared spectroscopy (nirs) data suggests a global circulatory origin for low-frequency oscillation signals in human brain. Neuroimage 53, 553–564. 10.1016/j.neuroimage.2010.06.049 20600975 PMC3133965

[B69] Toppi J. Borghini G. Petti M. He E. J. De Giusti V. He B.. (2016). Investigating cooperative behavior in ecological settings: an eeg hyperscanning study. PLoS ONE 11:e0154236. 10.1371/journal.pone.0154236 27124558 PMC4849782

[B70] Toronov V. Webb A. Choi J. H. Wolf M. Michalos A. Gratton E.. (2001). Investigation of human brain hemodynamics by simultaneous near-infrared spectroscopy and functional magnetic resonance imaging. Med. Phys. 28, 521–527. 10.1118/1.1354627 11339749

[B71] Torrence C. Compo G. P. (1998). A practical guide to wavelet analysis. Bull. Am. Meteorol. Soc. 79, 61–78.

[B72] Tsunashima H. Yanagisawa K. (2009). Measurement of brain function of car driver using functional near-infrared spectroscopy (fnirs). Comput. Intell. Neurosci. 2009:164958 10.1155/2009/164958 PMC2703809 19584938

[B73] van den Heuvel M. P. Sporns O. (2013). Network hubs in the human brain. Trends Cogn. Sci. 17, 683–696. 10.1016/j.tics.2013.09.012 24231140

[B74] Wickens C. D. Alexander A. L. (2009). Attentional tunneling and task management in synthetic vision displays. Int. J. Aviat. Psychol. 19, 182–199. 10.1080/10508410902766549

[B75] Wright N. McGown A. (2001). Vigilance on the civil flight deck: incidence of sleepiness and sleep during long-haul flights and associated changes in physiological parameters. Ergonomics 44, 82–106. 10.1080/00140130150203893 11214900

[B76] Xiong J. Parsons L. M. Gao J.-H. Fox P. T. (1999). Interregional connectivity to primary motor cortex revealed using mri resting state images. Hum. Brain Mapp. 8, 151–156. 10524607 10.1002/(SICI)1097-0193(1999)8:2/3<151::AID-HBM13>3.0.CO;2-5 PMC6873334

[B77] Zander T. O. Kothe C. (2011). Towards passive brain–computer interfaces: applying brain–computer interface technology to human–machine systems in general. J. Neural Eng. 8:025005. 10.1088/1741-2560/8/2/025005 21436512

[B78] Zander T. O. Kothe C. Jatzev S. Gaertner M. (2010). Enhancing human-computer interaction with input from active and passive brain-computer interfaces, in Brain-Computer Interfaces (Cham: Springer), 181–199.

[B79] Zhang X. Noah J. A. Hirsch J. (2016). Separation of the global and local components in functional near-infrared spectroscopy signals using principal component spatial filtering. Neurophotonics 3:015004. 10.1117/1.NPh.3.1.015004 26866047 PMC4742567

